# Defining the acute BTK-loss transcriptional program in CLL using a BTK degrader

**DOI:** 10.1038/s41375-026-02926-1

**Published:** 2026-03-20

**Authors:** Mingma G. Sherpa, Sutapa Sinha, Weiguo Han, Heather C. Darby, Sameer A. Parikh, Neil E. Kay, Zhiquan Wang

**Affiliations:** 1 Division of Hematology, Department of Medicine, Mayo Clinic, Rochester, MN USA; 2 Department of Immunology, Mayo Clinic, Rochester, MN USA

**Keywords:** Chronic lymphocytic leukaemia, Chronic lymphocytic leukaemia

## To the Editor

Constitutive activation of the B-cell receptor (BCR) signaling pathway is a hallmark of chronic lymphocytic leukemia (CLL), driving the malignant cell survival, proliferation, and interaction with the tumor microenvironment [1]. Over the past decade, inhibition of BCR signaling by small-molecule inhibitors targeting Bruton tyrosine kinase (BTK), such as ibrutinib, acalabrutinib, and zanubrutinib, has transformed the treatment of CLL, producing durable responses in the majority of patients and fundamentally reshaping therapeutic algorithms [2]. Despite these advances, relapse and resistance remain inevitable. Both genetic and adaptive mechanisms contribute to therapeutic failure, including acquired mutations in *BTK* or *PLCG2* as well as broader rewiring of signaling and transcriptional networks [3]. To better understand mechanisms underlying BTKi response and resistance, it is important to define the immediate transcriptional consequences of BTK loss in primary CLL cells. Yet these effectors remain poorly defined, partly because most available data derive from steady-state expression analyses under conditions of chronic inhibition, which obscure immediate signaling outputs.

This gap in knowledge is further complicated by the biology of BTK itself. In addition to its well-characterized enzymatic role in propagating BCR signaling, BTK also participates in kinase-independent scaffolding interactions [4]. Conventional ATP-competitive inhibitors, such as ibrutinib, only block catalytic activity while leaving non-catalytic functions intact. Consequently, studies relying on such inhibitors may fail to capture the full scope of BTK activity. Moreover, prolonged inhibition permits compensatory adaptations that reshape transcriptional programs. As a result, the acute transcriptional programs regulated by BTK in primary CLL cells remain incompletely defined, limiting mechanistic insight into BTK-dependent signaling and therapeutic response.

To address this challenge, we leveraged NX-5948, an orally bioavailable small-molecule degrader of BTK [5]. Unlike inhibitors, NX-5948 induces rapid proteasomal degradation of BTK, eliminating both kinase-dependent and kinase-independent functions. Its acute activity reduces the influence of secondary or compensatory responses, enabling us to capture the immediate transcriptional consequences of BTK loss. This strategy is conceptually similar to recent efforts to map oncogene-dependent transcriptional programs in solid tumors, such as the definition of KRAS- and ERK-dependent gene sets in lung cancer [6]. Accordingly, we used NX-5948 as a tool to define an acute BTK-loss transcriptional signature in primary CLL cells.

**Fig. 1:**
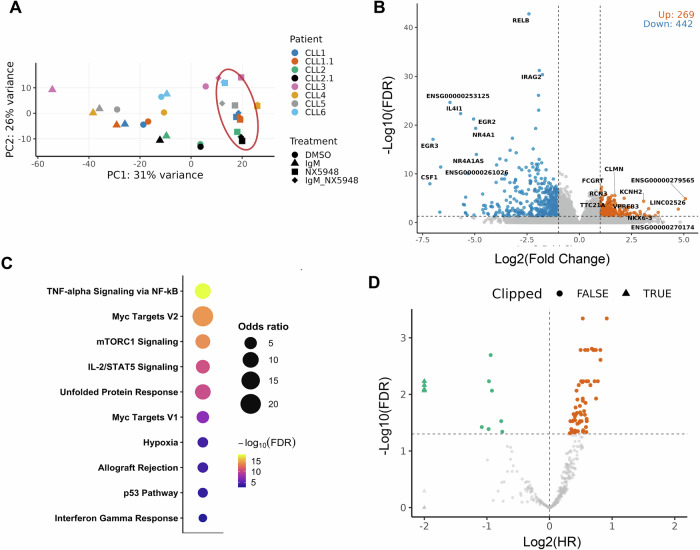
NX-5948 defines a BCR-dependent transcriptional signature with clinical relevance in CLL. **A** Supervised Principal component analysis (PCA) for gene expression profiles of CLL B cells treated with DMSO, anti-IgM, NX-5948, or anti-IgM+NX-5948 across six independent donors. Only the genes consistently differentially expressed between anti-IgM+NX-5948 vs anti-IgM were used. CLL1.1 and CLL1.2 are replicates of CLL1 and CLL2. **B** Volcano plot of differential expression following NX-5948 treatment. A total of 269 genes were significantly upregulated and 442 downregulated (FDR < 0.05, log₂ fold change > 1). Selected genes with strong significance are labeled. **C** Gene set enrichment analysis of NX-5948-downregulated transcripts revealed suppression of hallmark oncogenic and stress-adaptive pathways. **D** Clinical relevance of NX-5948-regulated genes was evaluated using the ICGC CLL dataset. Higher expression of 67 NX-5948-downregulated genes was significantly associated with worse overall survival (OS), while higher expression of 9 was associated with better OS (FDR < 0.05).

We treated CLL cells from six independent untreated patients with NX-5948, anti-IgM, or vehicle control, and performed bulk RNA sequencing (Supplementary Fig. 1A, B, Supplementary Table 1). Although the sample size is limited, this cohort represents broad and key aspects of CLL clinical heterogeneity, including FISH abnormalities (13q − , 11q − , 17p − , trisomy 12, and FISH-negative cases) and IGHV mutation status (3 mutated and 3 unmutated). Principal component analysis confirmed that inter-patient variability explained the majority of global transcriptional variance (Supplementary Fig. 1C), consistent with the genetic and clinical heterogeneity of CLL [7]. Nevertheless, NX-5948 exposure induced a consistent secondary axis of separation across all donors, reflecting reproducible transcriptional effects despite background heterogeneity (Fig. 1A). Differential expression analysis identified 269 significantly upregulated and 442 significantly downregulated transcripts (false discovery rate (FDR) < 0.05; |log₂ fold change | > 1) (Fig. 1B, Supplementary Fig. 1D and Supplementary Table 2). Many of the top-downregulated genes (e.g., RELB, EGR2, CSF1, NR4A1) play important roles in CLL pathogenesis. Pathway-level analysis of these transcriptional changes revealed that NX-5948 suppresses multiple hallmark oncogenic programs downstream of BCR signaling. Among the most significantly downregulated were TNF-α signaling via NF-κB, MYC target genes, mTORC1 signaling, IL-2/STAT5 signaling, the unfolded protein response, hypoxia responses, the p53 pathway, and interferon-γ signaling (Fig. 1C, Supplementary Table 3). These pathways have each been implicated in CLL biology and therapeutic resistance, supporting their regulation downstream of BTK signaling. We thus define this set of genes as an acute BTK-loss induced transcriptional signature in CLL. Notably, the upregulated genes did not show significant enrichment in known pathways associated with CLL biology (Supplementary Table 4).

We further examined the clinical relevance of these transcriptional changes using the published ICGC dataset [8, 9]. Of the 442 NX-5948-downregulated genes, 391 were annotated and expressed in this dataset. Higher expression of 67 of these genes was significantly associated with worse overall survival (OS) (HR > 1, FDR < 0.05), whereas higher expression of 9 genes was associated with improved OS (Fig. 1D). In contrast, among the NX-5948-upregulated genes, higher expression of 8 genes was linked to better OS and 7 genes to worse OS (Supplementary Fig. 1E). These findings indicate that genes suppressed by BTK degradation are strongly enriched for clinically relevant outcome associations in CLL.

To determine whether NX-5948–induced transcriptional changes are BTK-specific or reflect broader kinase inhibition, we compared our data with RNA-seq from primary CLL B cells treated with ibrutinib for 24 h (Supplementary Fig. 1F). We identified 173 genes commonly downregulated by both treatments, 269 genes uniquely downregulated by NX-5948, and 319 genes uniquely downregulated by ibrutinib (Supplementary Fig. 1G). Pathway enrichment analysis showed significant and overlapping pathway enrichment for the NX-5948–specific and shared gene sets, whereas pathway enrichment among ibrutinib-specific genes was weak or not significant (Supplementary Fig. 1H), suggesting that NX-5948 more selectively captures BTK-dependent transcriptional programs involved in CLL pathobiology.

**Fig. 2:**
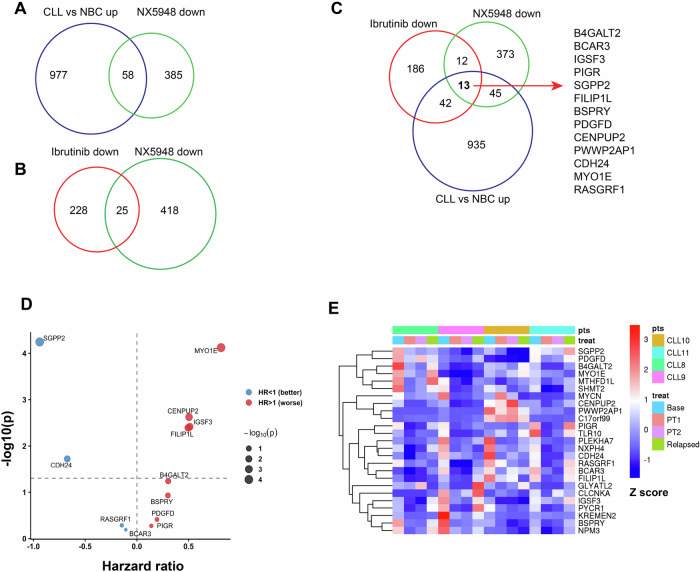
NX-5948-downregulated genes associate with therapy-responsive signatures in CLL. **A** Venn diagram showing overlap between NX-5948-downregulated genes and those upregulated in CLL versus normal B cells (NBC). **B** Venn diagram showing overlap between NX-5948-downregulated genes and transcripts suppressed after one year of ibrutinib therapy. **C** Triple overlap analysis identifies 13 shared genes between CLL vs NBC-up, NX-5948-down, and ibrutinib-down signatures; representative genes are listed. **D** Survival analysis of the 13 shared genes in the ICGC dataset shows that several genes (red) were associated with worse overall survival (HR > 1). **E** Heatmap of overlapping genes across independent CLL cohorts demonstrates consistent expression changes in baseline, on-treatment, and relapsed samples.

To further assess clinical relevance, we compared this signature to disease and therapy-associated expression patterns. First, we compared the gene expression between CLL B cells and normal B cells (NBC) (n = 6 for each group) (Supplementary Fig. 2A) [10]. We identified 58 genes upregulated in CLL compared with NBC, which were also downregulated upon NX-5948 treatment (Fig. 2A, Supplementary Table 5). These genes likely represent disease-associated transcriptional programs actively sustained by BCR signaling. Consistent with this, several of these genes are linked to oncogenic signaling pathways (Supplementary Fig. 2B). Second, we intersected NX-5948-regulated genes with longitudinal expression profiles from CLL patients (*n* = 12) treated with ibrutinib [11]. Twenty-five genes downregulated after one year of BTKi therapy were also suppressed by 24 h’ NX-5948 treatment (Fig. 2B, Supplementary Table 6). Notably, 13 genes were common across all three contexts—upregulated in CLL versus normal B cells, downregulated by acute NX-5948 treatment, and downregulated after prolonged ibrutinib therapy (Fig. 2C). These overlapping genes represent candidate markers linked to BTK-dependent disease biology. We examined the clinical relevance of these 13 genes using the published ICGC dataset and found 4 genes (MYO1E, CENPUP2, IGSF3, and B4GALT2) were significantly associated with worse OS and 2 were associated with better OS (Fig. 2D, Supplementary Fig. 1C). We further analyzed the RNA-seq data from four CLL patients in our clinic who relapsed with ibrutinib treatment [11] and found that the expression of 7 of the 13 overlapped genes in the relapsed samples was restored toward the baseline levels in 3 patients (Fig. 2E). These findings suggest that a subset of these genes may be linked to BTKi induced clinical response and resistance in CLL.

We validated the downregulation of several overlapping genes at the protein level by western blot (Supplementary Fig. 2D). Notably, pathway enrichment analysis revealed that genes suppressed by BTK loss were significantly enriched for MYC target gene programs (Fig. 1C). Consistent with this observation, BTK depletion resulted in reduced MYC protein levels, as confirmed by western blot, supporting a role for MYC as a downstream mediator of BTK-dependent transcriptional regulation.

In conclusion, NX-5948 defines an acute BTK-loss transcriptional signature in primary CLL that captures the immediate transcriptional consequences of BTK depletion. Although the number of primary samples is limited, the cohort reflects the known spectrum of clinical heterogeneity in CLL, including diverse FISH abnormalities and IGHV mutation status. Despite substantial inter-patient variability, we identified a reproducible set of differentially expressed genes using stringent criteria. We note that acute degradation may also induce secondary stress responses that contribute to a subset of observed transcriptional changes. Future studies in larger cohorts and across distinct BTK-targeting strategies will further refine this BTK-loss signature.

The BTK-loss signature includes hallmark oncogenic programs and overlaps with disease-associated and BTKi-responsive transcriptional changes observed in patients, supporting its biological and clinical relevance. Although the set of 13 genes shared with longitudinal ibrutinib treatment is biologically heterogeneous, several have plausible links to CLL pathobiology. For example, our previous work demonstrated that PDGFD promotes microenvironmental signaling in CLL and contributes to stromal-mediated support of leukemic cell survival [12]. RASGRF1, a Ras guanine nucleotide exchange factor, may connect BTK loss to reduced Ras/MAPK–ERK pathway activity, consistent with evidence that BCR-driven ERK signaling and Ras–MAPK alterations contribute to CLL biology and therapeutic adaptation [13]. MYO1E, which regulates migration of activated B cells to lymph nodes, suggests a potential link between BTK depletion and altered leukemic cell trafficking or niche interactions [14]. Conversely, overlap genes without an established BTK/CLL connection may reflect indirect consequences of acute BTK loss, including MYC-linked transcriptional reprogramming and/or stress- or context-dependent responses. Subsequent experimental validation of these genes will be important to clarify their roles in CLL biology and to assess their potential as therapeutic targets. Future systematic comparisons across additional BTK inhibitors and degraders, as well as complementary datasets such as phosphoproteomics and epigenetic profiling, will further refine BTK-dependent regulatory networks. More broadly, this work highlights the utility of degrader technologies as mechanistic tools to dissect oncogenic signaling pathways and advance translational research in hematologic malignancies.

## Supplementary information

SUPPLEMENTAL data and method

Supplementary_table1

Supplementary_table2

Supplementary_table3

Supplementary_table4

Supplementary_table5

Supplementary_table6
