# Supplementary material for: Defining the acute BTK-loss transcriptional program in CLL using a BTK degrader
Source: Leukemia. 2026 Mar 20;40(6):1314–7. doi: 10.1038/s41375-026-02926-1 (PMC13233297; doi:10.1038/s41375-026-02926-1)
Supplement: Supplementary file 1 — SUPPLEMENTAL data and method [file 41375_2026_2926_MOESM1_ESM.docx]

**Defining the BCR-dependent transcriptional signature in chronic lymphocytic leukemia**

Mingma G. Sherpa^1^, Sutapa Sinha^1^, Weiguo Han^1^, Heather C. Darby^1^, Sameer A. Parikh^1^, Neil E. Kay^1,2^, Zhiquan Wang^1^

1, Division of Hematology, Department of Medicine, Mayo Clinic, Rochester, MN, 55905, USA.

2, Department of Immunology, Mayo Clinic, Rochester, MN, 55905, USA.

Correspondence: Zhiquan Wang, [wang.zhiquan@mayo.edu](mailto:wang.zhiquan@mayo.edu) or Neil E. Kay, [kay.neil@mayo.edu](mailto:kay.neil@mayo.edu)

**Supplementary Materials**

**This file includes**:

- Description of supplementary Tables
- Supplementary Figures 1 to 2
- Materials and Methods
- References for Supplementary Materials and Methods

**Description of supplementary Tables**

**Supplementary Table 1.** Clinical and biological characteristics of the CLL patient samples used in this study.

**Supplementary Table 2.** Differential expression results for NX-5948–treated versus control CLL cells.
**Supplementary Table 3.** Hallmark pathway enrichment results for genes downregulated by NX-5948.

**Supplementary Table 4.** Hallmark pathway enrichment results for genes upregulated by NX-5948.

**Supplementary Table 5.** Overlapping genes between genes upregulated in CLL versus NBC and genes downregulated in NX-5948-treated versus control samples.

**Supplementary Table 6.** Overlapping genes between genes downregulated after longitudinal ibrutinib treatment and genes downregulated in NX-5948-treated versus control samples.


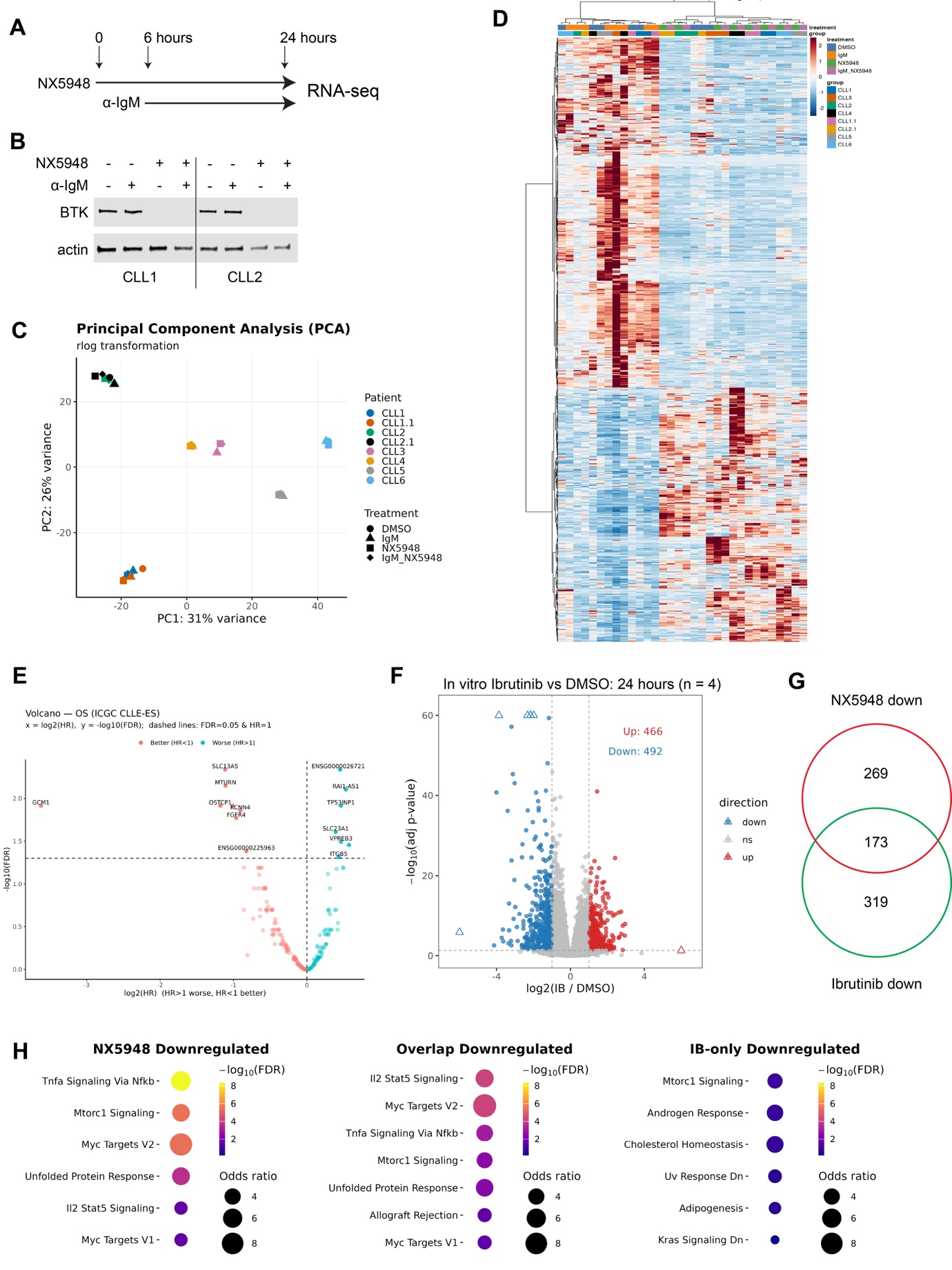


**Supplementary Figure 1. Experimental design, BTK degradation, and transcriptional consequences of NX-5948 in CLL.**
(A) Schematic of *in vitro* treatment timeline: CLL cells were exposed to NX-5948 for 24 hours, with α-IgM stimulation added 6 hours after NX-5948.

(B) Immunoblot showing efficient BTK degradation in two representative CLL donors following NX-5948 treatment with or without IgM stimulation; β-actin was used as loading control.

(C) PCA of rlog-transformed RNA-seq profiles across six healthy donors shows consistent separation of samples based on donors. CLL1.1 and CLL1.2 are replicates of CLL 1 and CLL2.

(D) Heatmap of differentially expressed genes across donors and treatment conditions highlights reproducible transcriptional suppression by NX-5948.

(E) Survival analysis of NX-5948-regulated genes using the ICGC CLL dataset identifies several transcripts significantly associated with overall survival (FDR < 0.05); red indicates higher expression linked to worse outcome (HR > 1), blue to better outcome (HR < 1).

(F) Volcano plot showing differentially expressed genes in primary CLL B cells treated *in vitro* with ibrutinib versus DMSO for 24 hours (n = 4). CLL cells were exposed to ibrutinib for 24 hours, with α-IgM stimulation added 6 hours after ibrutinib. Genes are colored by direction of change (upregulated, downregulated, or not significant). Dashed lines indicate significance thresholds.

(G) Venn diagram showing overlap between genes significantly downregulated by acute NX-5948 treatment and those downregulated by 24-hour ibrutinib exposure. Numbers indicate NX-5948–specific, shared, and ibrutinib-specific downregulated genes.

(H) Pathway enrichment analysis of downregulated gene sets. Left: genes uniquely downregulated by NX-5948; middle: genes commonly downregulated by both NX-5948 and ibrutinib; right: genes downregulated only by ibrutinib. Dot size reflects the odds ratio, and color indicates −log₁₀(FDR). NX-5948–specific and overlapping gene sets show enrichment for BTK-associated oncogenic pathways, whereas pathways enriched among ibrutinib-only genes are weaker and less consistent with associations to any pathway implicated in CLL pathobiology.

**
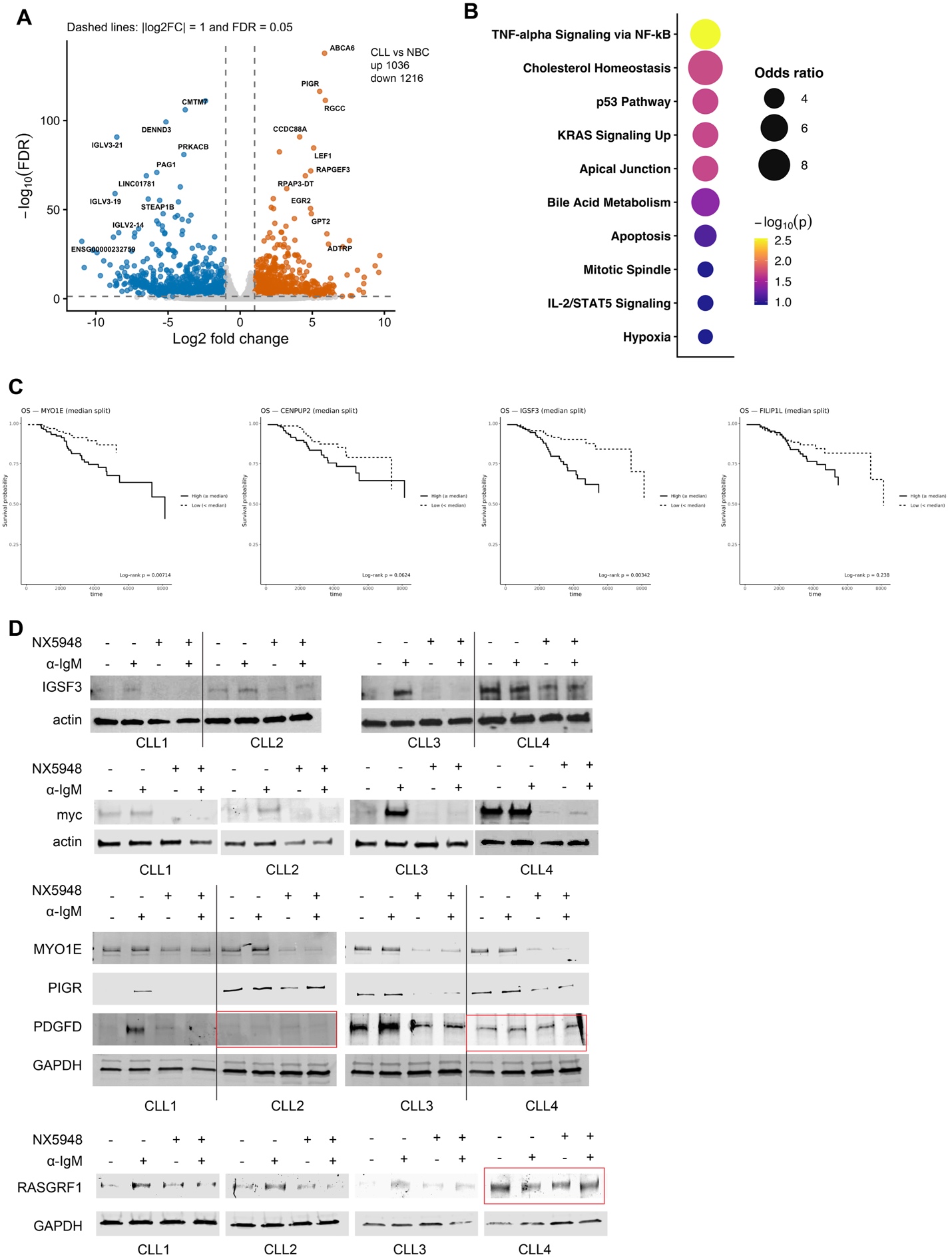
**

**Supplementary Figure 2. Association of NX-5948 downregulated genes with ibrutinib-related gene expression and CLL OS.**
(A) Volcano plot of differential expression between CLL and normal B cells identifies 1,036 upregulated and 1,216 downregulated transcripts (FDR < 0.05, |log₂FC| > 1); selected significant genes are labeled.

(B) Gene set enrichment analysis of overlapped NX-5948 downregulated and ibrutinib downregulated genes.

(C) Kaplan–Meier survival curves (ICGC dataset) for representative genes (MYO1E, CENPUP2, IGSF3, and FILIP1L) show associations between high expression and reduced overall survival.

(D) Immunoblot analysis of selected proteins encoded by genes within the acute BTK-loss transcriptional signature in primary CLL cells. Cells from four CLL donors (CLL1–CLL4) were treated with NX-5948, anti-IgM, or the combination, as indicated. Protein levels of IGSF3, MYC, MYO1E, PIGR, PDGFD, and RASGRF1 were assessed by western blot, with actin or GAPDH as loading controls. For most targets, protein changes were concordant with RNA-seq results. Red boxes indicate instances in which protein-level changes were variable or not fully concordant with transcript-level regulation, highlighting donor-specific heterogeneity and potential post-transcriptional regulation in primary CLL cells.

**Materials and Methods**

**Purification of leukemic and normal B cells.** All patients provide research blood samples by signing and submitting written informed consent according to the Declaration of Helsinki to the Mayo Clinic Institutional Review Board, which approved these studies. Informed consent was also obtained from healthy donors to obtain their B cells. The relevant patient information is shown in **Supplementary Table 1.** Primary CLL B cells and normal B cells were purified from blood samples using the RosetteSep B cell enrichment kit (Stem Cell Technologies). The typical purification of CD5+/CD19+ CLL B cells was > 95%, as determined by flow cytometric analysis. Normal B cells were purified from blood samples using the RosetteSep B cell enrichment kit (Stem Cell Technologies) with CD19+ CLL B cells at> 95%.

**Cell culture and treatment**. CLL B cells were cultured in AIM V medium with NX-NX-5948 (MedChemExpress) (1nM) or DMSO for 6 hours. Then, the anti-IgM (10 μg/ml) or vehicle control was added to the culture for another 18 hours before the cells were harvested.

**RNA-seq.** Total RNA was extracted using the Direct-zol RNA Kit (Zymo Research). Library preparation and sequencing were performed using the NovaSeq 6000 platform, paired-end 150 bp by Novogene (Sacramento, CA).

**RNA-seq data analysis**. Reads were aligned using Star^1^. Transcript abundance files were then used in the DESeq2 R package, which was used for all downstream differential expression analysis and generation of volcano plots. Differentially expressed genes between samples from healthy individuals and CLL patients were compared with a cutoff of fold change > 2, FDR <0.05.

**Western blotting**. Whole cell lysates were extracted in Laemmli buffer (60 mM Tris-Cl pH 6.8, 2% SDS, 10% glycerol, 5% β-mercaptoethanol, 0.01% bromophenol blue), separated by SDS-PAGE gel, and transferred to PDVF membrane (MilliporeSigma). Blots were blocked using Odyssey Blocking Buffer (LI-COR Biosciences, Lincoln, NE) prior to incubation with primary antibody at 4°C overnight. Secondary antibody incubations (anti-mouse, 1:5,000 dilution; anti-rabbit, 1:10,000 dilution; both LI-COR Biosciences) were performed for 1 hour at room temperature. Proteins of interest were visualized by Odyssey infrared imaging system (LI-COR Biosciences). The following primary antibodies were used: BTK (D3H5) Rabbit mAb (Cell Signaling Technology, #8547), MYO1E (Proteintech, #17768-1-AP), PDGFD (Proteintech, #14075-1-AP), PIGR (Abcam, #ab96196), RASGRF1 (Proteintech, #12958-1-AP), c-Myc (Abcam, #ab32072).

**ICGC survival analysis**. We analyzed the International Cancer Genome Consortium (ICGC) chronic lymphocytic leukemia cohort (CLLE-ES) with available baseline bulk-tumor transcriptomes and overall-survival (OS) metadata. Gene identifiers were harmonized to HGNC symbols. Expression values were log₂-transformed when required and standardized to z-scores within cohort prior to modeling. For each gene, we fit a Cox proportional-hazards model with continuous z-scored expression as the predictor and OS as the outcome; models were adjusted for age and sex when these covariates were present and informative (i.e., non-constant and with sufficient non-missing values). We report the hazard ratio (HR) per +1 SD increase in expression, 95% confidence interval (CI) from the Wald estimator, and nominal p value. Multiple testing was controlled at the gene level using Benjamini–Hochberg false-discovery rate (FDR). “Worse-outcome” genes were defined as HR>1 with 95% CI excluding 1 and FDR<0.05; “better-outcome” genes were defined analogously with HR<1, CI excluding 1, and FDR<0.05. For visualization, Kaplan–Meier curves were produced for flagged genes by dichotomizing expression at the cohort median (minimum 8 samples per arm), with log-rank p values shown for reference; continuous-effect estimates from Cox were used for inference.

**References**:

1. Dobin A, Davis CA, Schlesinger F, Drenkow J, Zaleski C, Jha S*, et al.* STAR: ultrafast universal RNA-seq aligner. *Bioinformatics* 2013 Jan 1; **29**(1)**:** 15-21.
